# Supplementary material for: IW-Scoring: an Integrative Weighted Scoring framework for annotating and prioritizing genetic variations in the noncoding genome
Source: Nucleic Acids Res. 2018 Jan 30;46(8):e47. doi: 10.1093/nar/gky057 (PMC5934661; doi:10.1093/nar/gky057)
Supplement: Supplementary Data [file gky057_supp.zip › nar-03663-met-n-2017-File008.pdf]

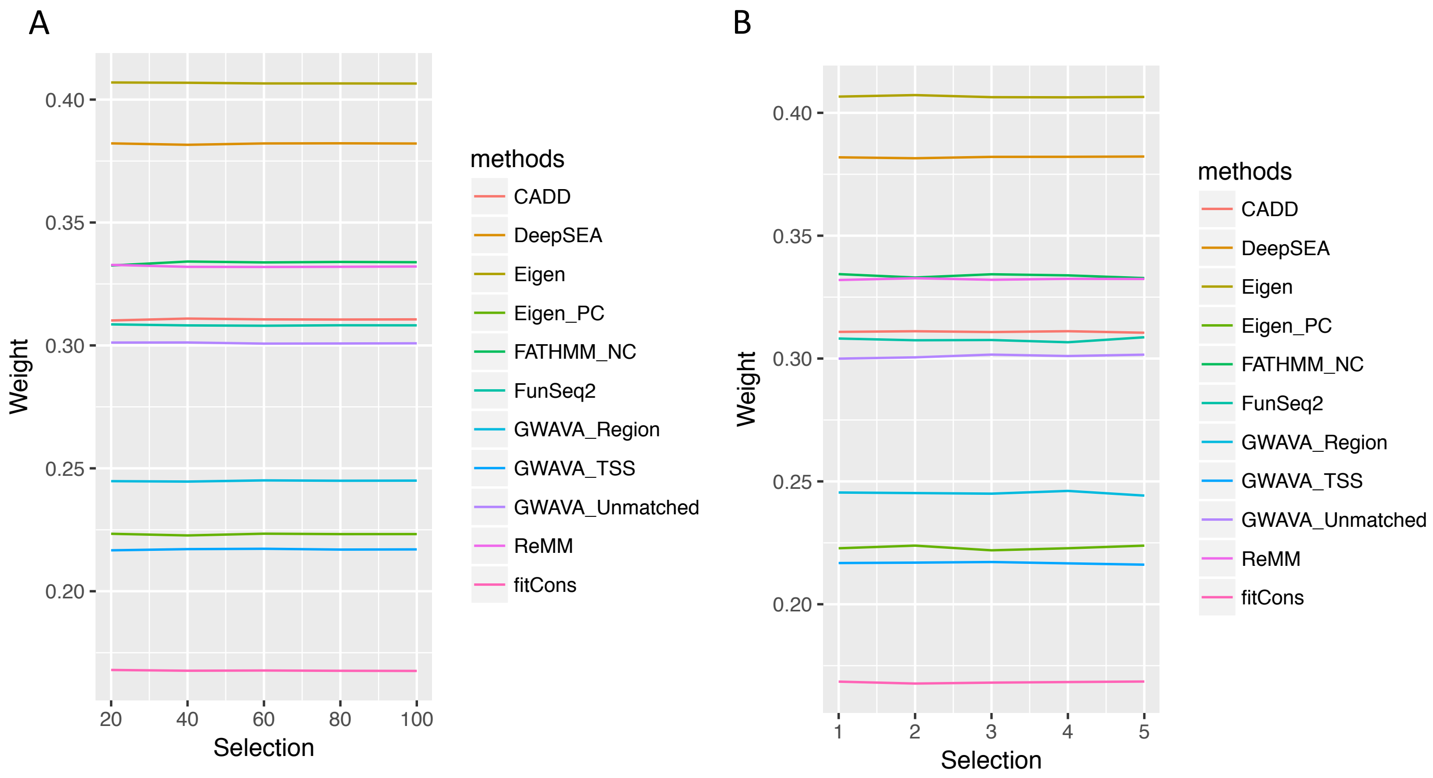

Supplementary Figure S1. Weight estimation for the 11 constituent scores by selecting, (A) a subset of training data set variants, 20%, 40%, 60% and 80%, and (B) 20% of training data set by 5 times.

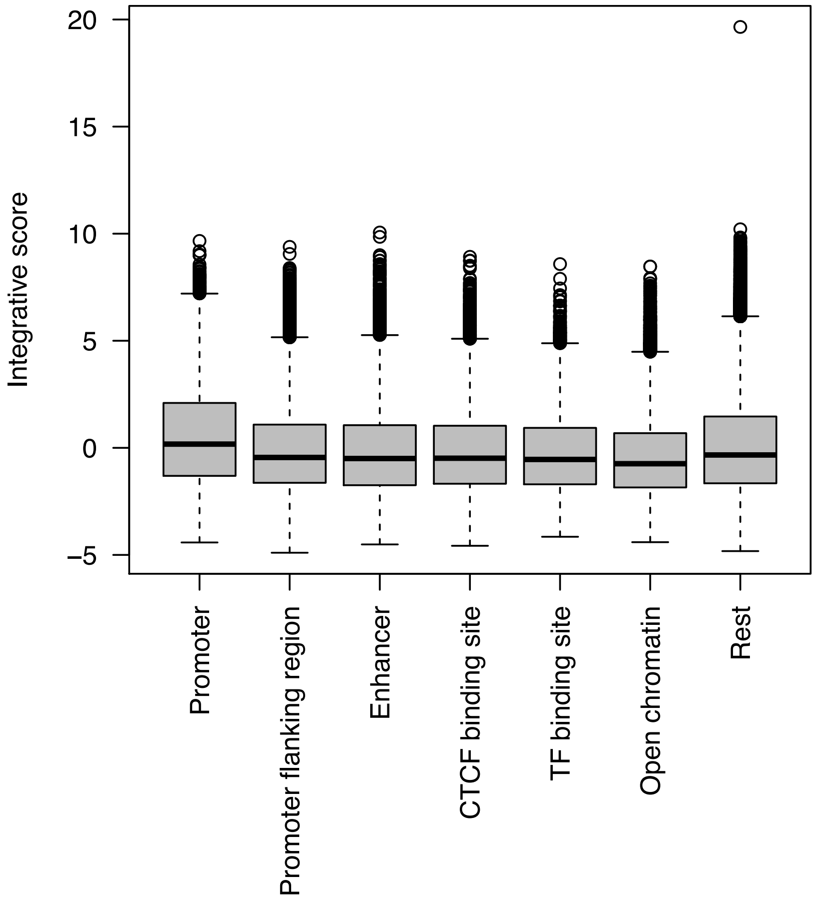

Supplementary Figure S2. Integrative scores for mutations within various Ensembl Regulatory Build annotation features for training variants.

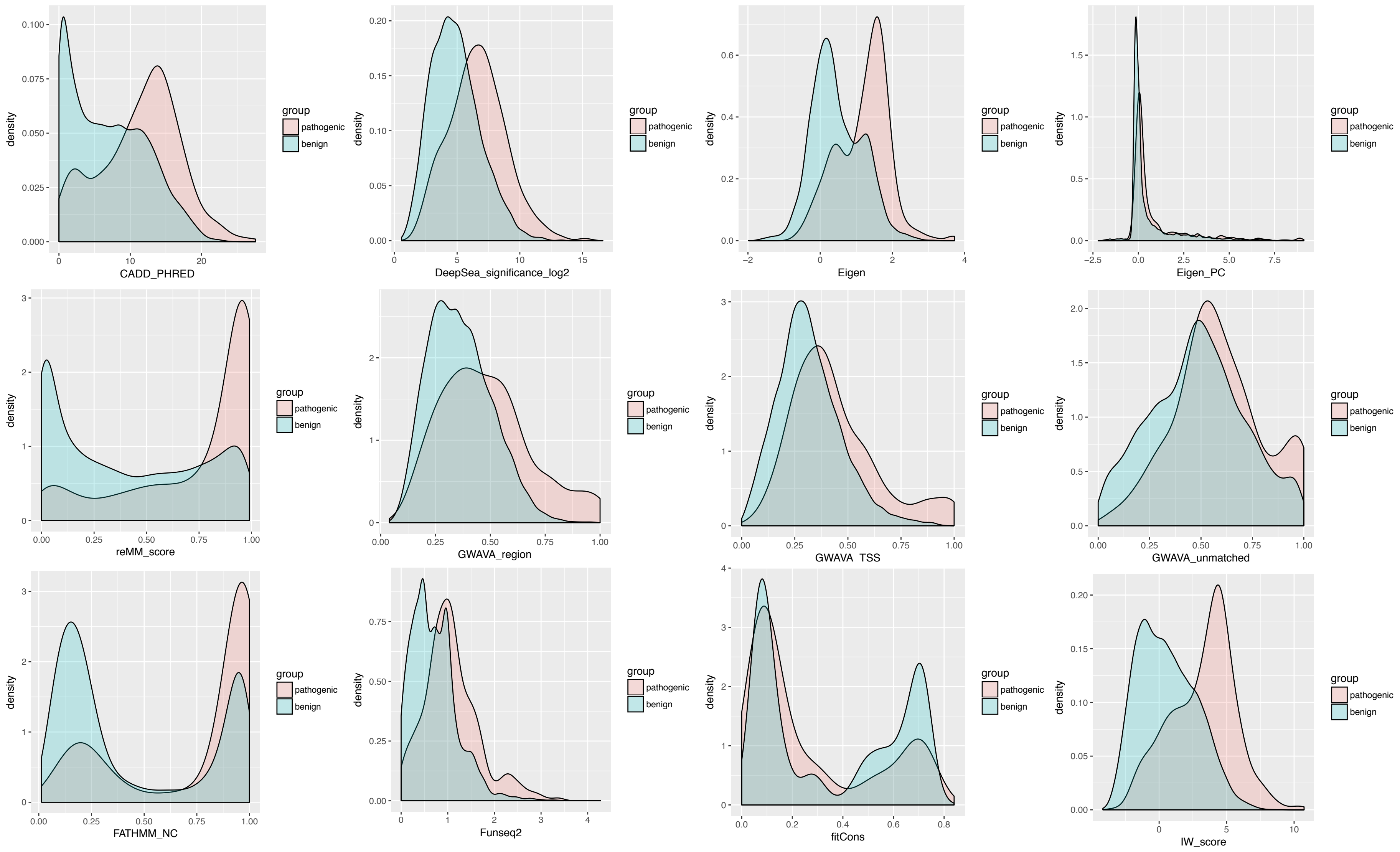

Supplementary Figure S3. Distribution of functional scores for selected ClinVar pathogenic and benign variants across all methods.

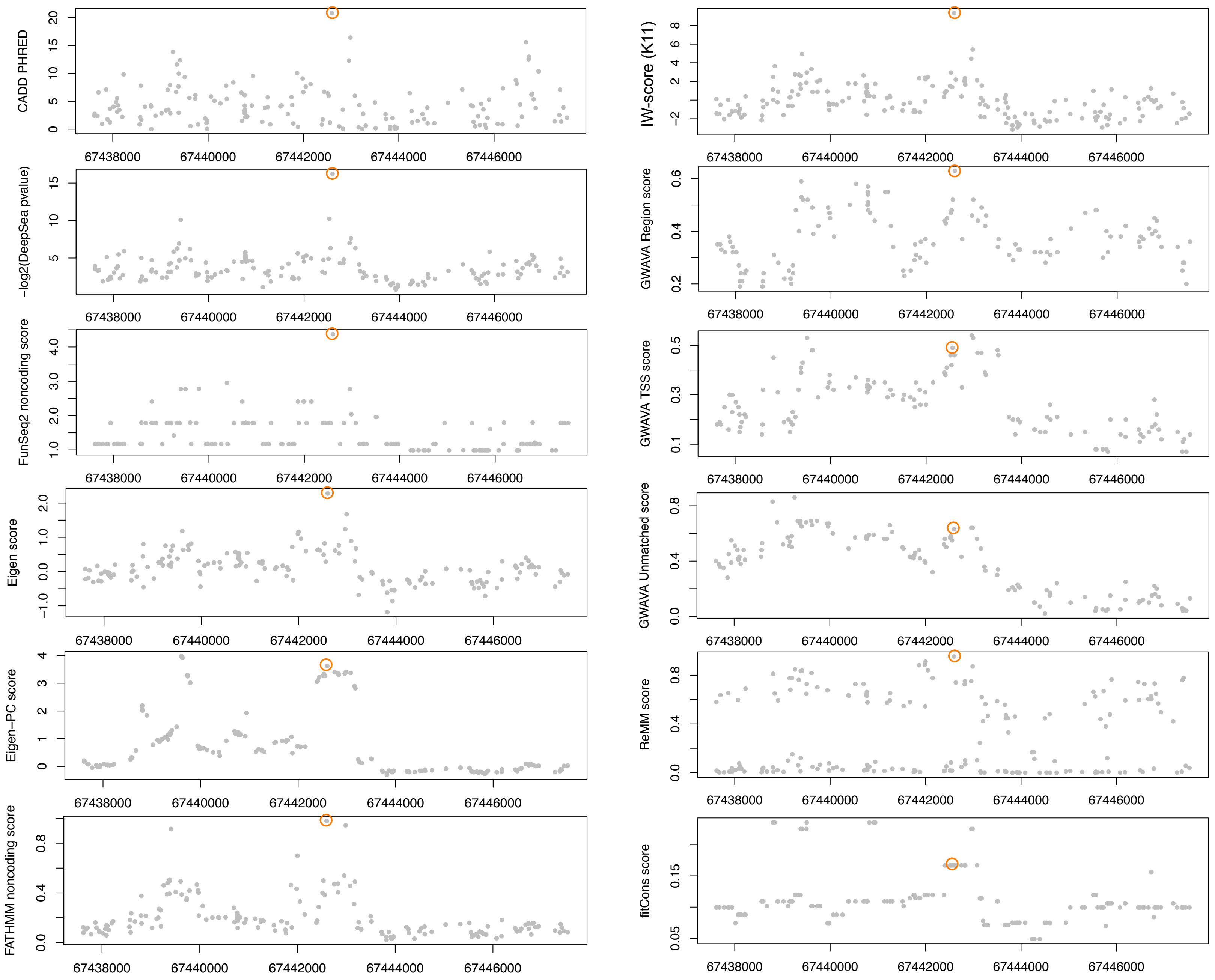

Supplementary Figure S4. Predicted functional score of rs17293632 along with the scores for all nearby known variants within 5 kb up- and downstream, for all other methods.

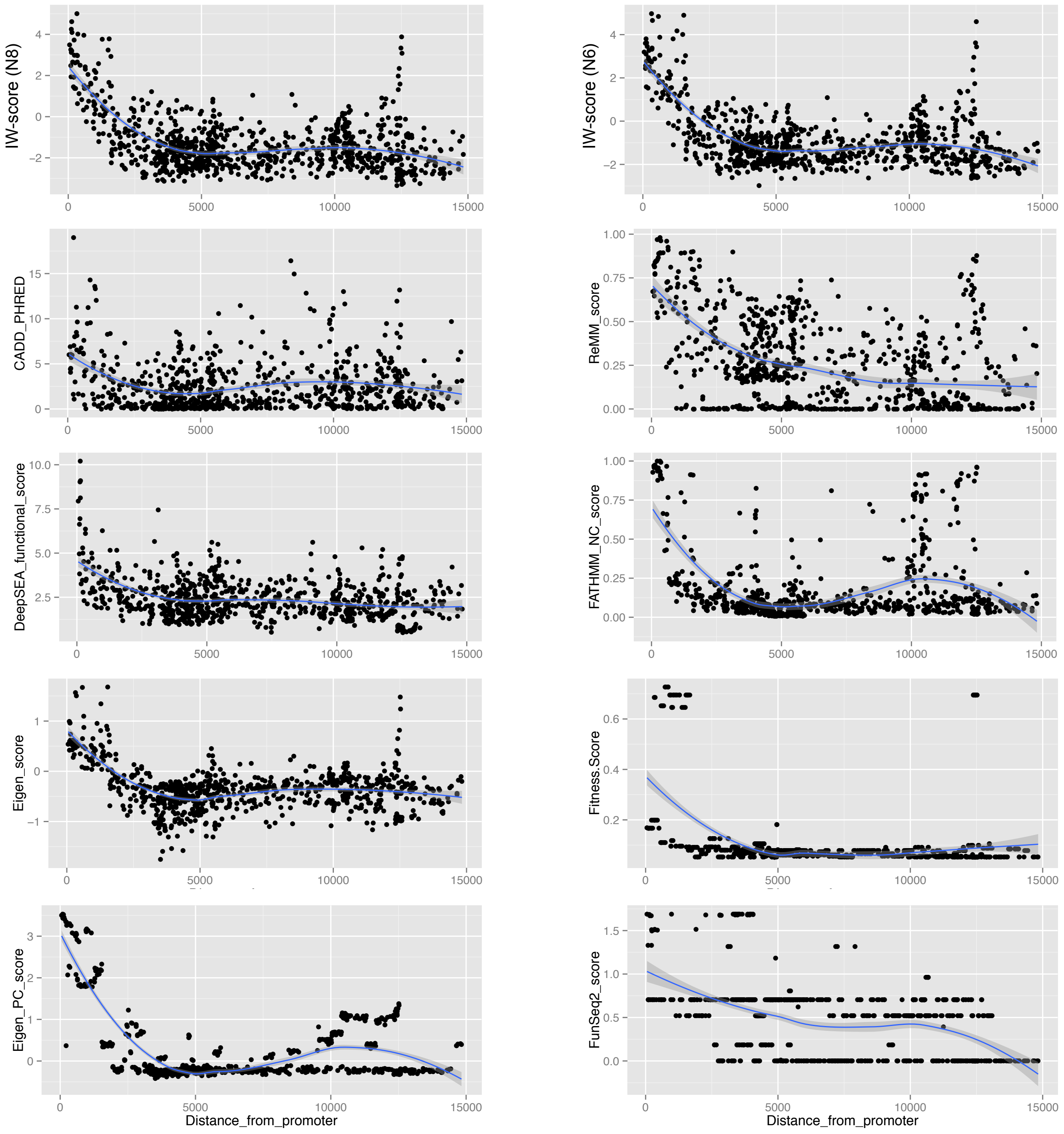

Supplementary Figure S5. Plots of functional scores against the distance to promoter for noncoding variants in and near *TERT* for all methods excluding GWAVA.

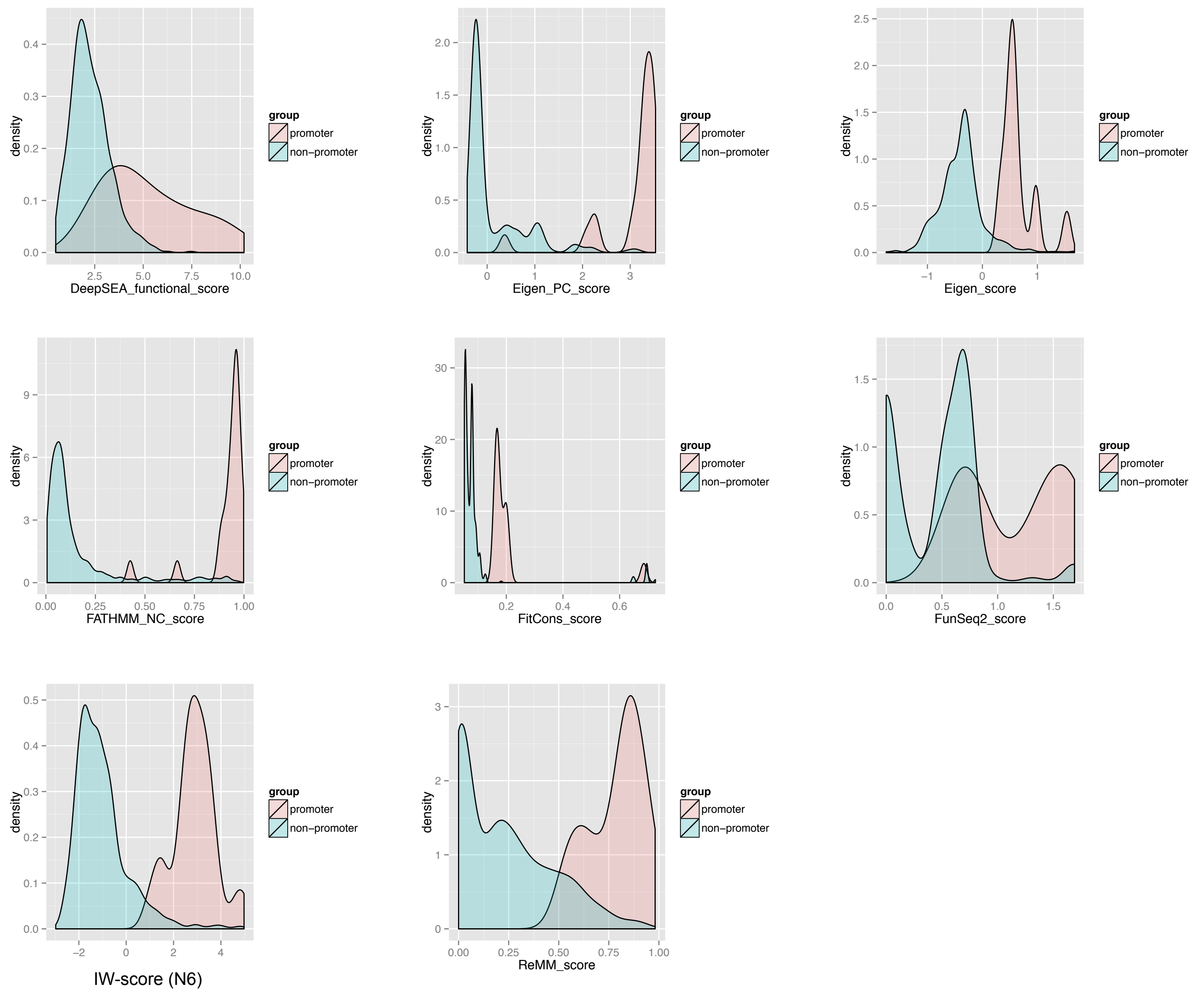

Supplementary Figure S6. Distribution of functional scores of noncoding variants in *TERT* promoter and non-promoter regions across different methods.

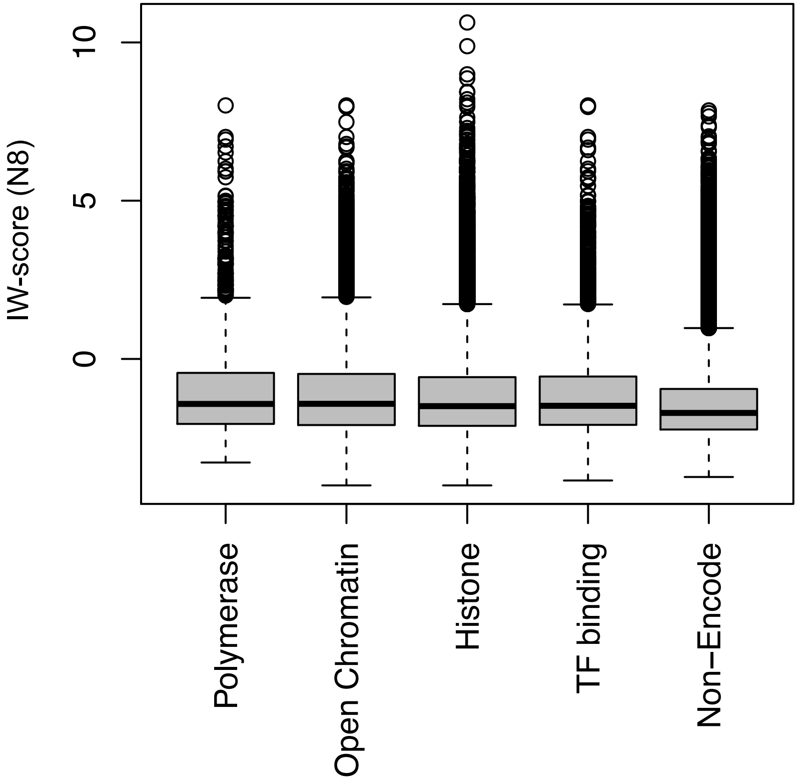

Supplementary Figure S7. Integrative scores, IW-score (N8), for mutations within various ENCODE annotated regions for FL noncoding mutations.

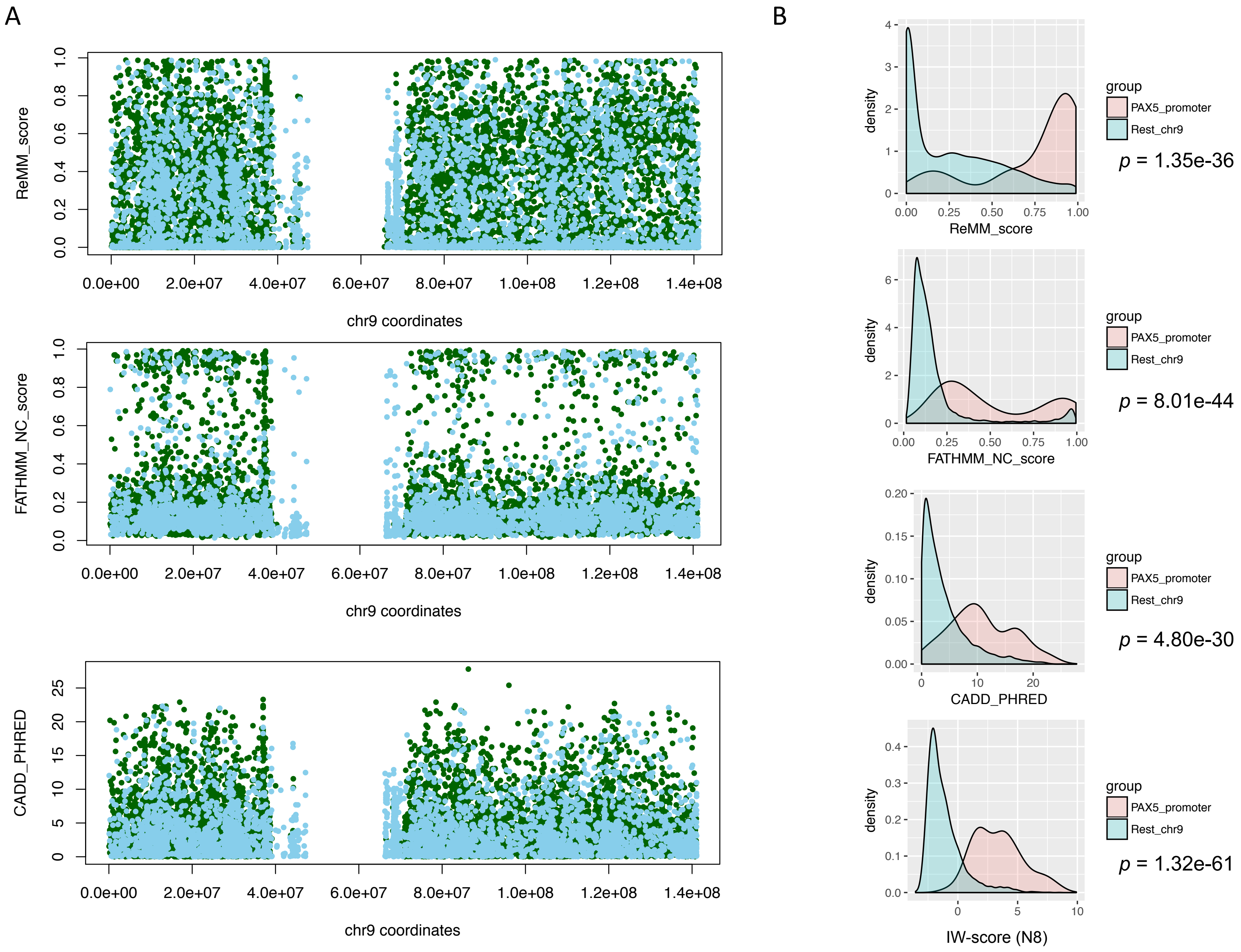

Supplementary Figure S8. Functional scores of noncoding mutations of FL in chr9 for ReMM, FATHMM noncoding and CADD PHRED scores. The differences in scores of noncoding mutations between *PAX5* promoter and first intron, and the rest of chr9, were also shown in panel B for selected methods, Wilcoxon test.
